# Investigation of Brain Activation Patterns Related to the Feminization or Masculinization of Body and Face Images across Genders

**DOI:** 10.3390/tomography8040176

**Published:** 2022-08-22

**Authors:** Carlo Ceruti, Alessandro Cicerale, Matteo Diano, Mattia Sibona, Caterina Guiot, Giovanna Motta, Chiara Crespi, Anna Gualerzi, Fabio Lanfranco, Mauro Bergui, Federico D’Agata

**Affiliations:** 1 Department of Surgical Sciences, Città della Salute e della Scienza Hospital, University of Turin, 10126 Torino, Italy; 2 CIDIGeM—Molinette Center for Gender Dysphoria Study Group, 10126 Torino, Italy; 3 Department of Neurosciences, University of Turin, 10126 Torino, Italy; 4 Department of Psychology, University of Turin, 10124 Torino, Italy; 5 Department of Endocrinology, Città della Salute e della Scienza Hospital, University of Turin, 10126 Torino, Italy; 6 Department of Psychology, Città della Salute e della Scienza Hospital, 10126 Torino, Italy; 7 Department of Psychiatry, Città della Salute e della Scienza Hospital, 10126 Torino, Italy

**Keywords:** fMRI, brain sex differences, visual face processing, visual body processing

## Abstract

Previous studies demonstrated sex-related differences in several areas of the human brain, including patterns of brain activation in males and females when observing their own bodies and faces (versus other bodies/faces or morphed versions of themselves), but a complex paradigm touching multiple aspects of embodied self-identity is still lacking. We enrolled 24 healthy individuals (12 M, 12 F) in 3 different fMRI experiments: the vision of prototypical body silhouettes, the vision of static images of the face of the participants morphed with prototypical male and female faces, the vision of short videos showing the dynamic transformation of the morphing. We found differential sexual activations in areas linked to self-identity and to the ability to attribute mental states: In Experiment 1, the male group activated more the bilateral thalamus when looking at sex congruent body images, while the female group activated more the middle and inferior temporal gyrus. In Experiment 2, the male group activated more the supplementary motor area when looking at their faces; the female group activated more the dorsomedial prefrontal cortex (dmPFC). In Experiment 3, the female group activated more the dmPFC when observing either the feminization or the masculinization of their face. The defeminization produced more activations in females in the left superior parietal lobule and middle occipital gyrus. The performance of all classifiers built using single ROIs exceeded chance level, reaching an area under the ROC curves > 0.85 in some cases (notably, for Experiment 2 using the V1 ROI). The results of the fMRI tasks showed good agreement with previously published studies, even if our sample size was small. Therefore, our functional MRI protocol showed significantly different patterns of activation in males and females, but further research is needed both to investigate the gender-related differences in activation when observing a morphing of their face/body, and to validate our paradigm using a larger sample.

## 1. Introduction

The ability to recognize our own body and face, and to differentiate them from bodies and faces belonging to other persons is a critical skill. While the neural correlates of the process of self-recognition have been studied extensively, there is less literature investigating the relationship between sex, gender, and bodily self-identity in modulating the activity of these areas in humans.

It has been argued [1] that our mechanisms of body and face recognition seem to rely on three main components: sensory perception of faces and bodies, the sensation of body ownership, and integration of the body into the concept of self. The neural substrates of body perception have been localized in a network of areas that include the extrastriate body area (EBA) and the fusiform body area (FBA). Interestingly, it seems that the role of the two areas in the processing of stimuli are not identical: while the EBA might be more activated during the perception of small body parts (such as hands or fingers) and might respond more intensely to movement, the FBA could be more specifically activated by holistic representations of the entire body [2], or possibly exhibit a “steplike” activation behaviour, selectively activating in response of large areas of the body, such as torsos or headless bodies [3]. Furthermore, the two areas might respond differently to motion, as it has been shown that the EBA might respond more to moving than to static body stimuli, while the same is not true for the FBA [3]. Regardless of their specific role in the neural perception of bodies, both EBA and FBA must be functionally integrated with other areas to carry out complex cognitive functions such as social perception or recognizing our own body [4]. In particular, the integration of EBA and FBA activity could be crucial in the recognition of the identity of the bodies (that is, the body of a person as distinct from the body of another person, regardless of factors such as scale or orientation of the stimuli [5]). It is not surprising then that both areas show a different activation between visual representations of own and other’s bodies [6].

The self vs. others discrimination is also carried out by areas such as the medial prefrontal cortex (mPFC). In particular, it seems that self-referential judgments activated the anterior mPFC, both in its ventral and dorsal areas [7], and that the ventral mPFC could be more activated by self-referential judgments characterized by higher emotive investment, rather than high degrees of certainty [8]. An imaging meta-analysis conducted using the Activation Likelihood Estimate approach [9] likewise finds clusters of activations linked to the self-others contrast in the right ventral mPFC. The same meta-analysis also identifies other areas characterized by a degree of self-specificity, such as the ventral and dorsal anterior cingulate cortex and the anterior insula. Like for the perception and processing of stimuli related to human bodies, the neural underpinnings of the perception of human faces and self-other facial discrimination have been localized in a network of areas: just as the FBA shows specific activation for human bodies, the fusiform face area (FFA) is activated by the sight of human faces [10], while an effect-location meta-analysis conducted on neuroimaging studies [11] investigating self-face recognition identified increased activity in areas such as the right precuneus and the left fusiform gyrus. Interestingly, the results of this meta-analysis agree with previous work [12] about the role of the precuneus, seen as the second step of a tripartite model, where the first stage is the sensory processing of the faces; the second stage processes self-referential facial information and the third stage (higher cortical substrates) are involved in identity discrimination tasks. As both face and body recognition are important elements of our self-image, it is not surprising that a meta-analysis [13] identified areas seems to be differentially activated by the self-other contrast both for faces and for bodies, such as the insula (and more specifically, the right insula), the anterior cingulate cortex and the inferior parietal lobule. Some of these results are confirmed by a more recent meta-analysis [14], which confirmed the role played by the ventral mPFC and by the ACC in self-face recognition tasks, as well as showing the role of the left parietal lobe and superior temporal gyrus.

The modulation of the activity in these areas, including the influence of different aspects such as sex and internal perception of oneself, is poorly understood. A recent study [1] found no differences between males and females in a task that contrasted images of their bodies to scrambled control images, but found gender-specific differences in other tasks. For instance, while female participants showed no differential brain activation when seeing bodies of other females as contrasted to their own, male participants did activate more the FBA, the left ventrolateral prefrontal cortex, and the left precentral gyrus, suggesting higher attentional and cognitive engagement for a self vs. others of the same sex. Other gender-related differences included the differential activation of the precuneus and right temporoparietal junction in a self vs. others of a different sex contrast: while men activated these areas more, women deactivated them. For males only, some areas were also more activated when seeing a picture of their body morphed towards the opposite sex: the precuneus, the amygdala and the caudate nucleus. This study suggests that self vs. others discrimination tasks can be influenced both by the gender of the subjects and the gender of the people represented in the stimuli. A further study from the same workgroup [15] expands on these findings, pointing out that gender identity (that is, the gender with which the participants self-identify), and not biological (assigned at birth) sex might be the relevant variable in defining the ‘same gender vs. different gender’, and highlighting the interaction between the dimensions at play in the self-identification with graphical representations of faces and bodies.

It is well known that fMRI studies have been extensively used in previous papers to investigate the difference between patterns of activation of males and females on a variety of cognitive tasks such as verbal fluency, mental rotation, and motivational tasks, [15,16,17,18]. However, to the best of our knowledge, no study replicated the approach shown in [1] using pictures of both faces and bodies, despite the overlap in the areas involved in the two tasks.

While Burke and collaborators used morphed pictures of the participants’ bodies, it is known that body-specific areas also respond to stick figures or silhouettes [19]. Using silhouettes instead could then allow investigating the gender-specific differences in body perception and self-identification, without the need for more realistic stimuli, and eliminating altogether the effect of confounding variables, such as BMI or height. On the other hand, using morphed faces to investigate face perception and processing is more common [20,21] especially when the tasks involve a self-other distinction. Furthermore, adding an experiment where the stimuli are composed of short clips of morphing faces rather than static photos could allow us to better identify the areas involved in the task, through the differential activation of areas involved in the processing of motion, also in the light of studies that found a dissociation between the processing of static and moving bodies and faces [2]. Therefore, this study The paradigm proposed in this paper aimed at specifically investigating processing streams that link self-perception and self-awareness with gender identity and sex of the stimuli, and at developing an fMRI protocol that could be used in further studies to investigate gender-related differences in self-identification. To do so, we realized a paradigm based on multiple tasks, as it is probably required to better define the blurred contours of gender-related differences in brain activation [22]. The tasks are characterized by reproducible manipulation of visual stimuli representing bodies and faces, and tested the paradigm on an explorative sample of male and female individuals, comparing the brain activity across genders. The areas of expected differential activation between males and females include areas that have been linked with self-other distinction and self-identification, such as the medial prefrontal cortex, precuneus and posterior cingulate cortex when observing stimuli representing bodies [23,24], as well as areas that are known to be sensitive to faces, such as the occipital face area, the fusiform face area, the temporal sulcus. Finally, as asymmetric brain activations have been noted in past studies [25], it is possible that such differences will follow such asymmetry. In the next section of the paper, we describe the approach we adopted, our main results and their discussion, with strengths and limitations.

## 2. Materials and Methods

### 2.1. Sample

Twenty-four healthy individuals were enrolled in this study: 12 males and 12 females, mean age 29 years (range 24–47), with self-reported gender identity matching the sex assigned at birth, heterosexual orientation, no present or past comorbidity assessed by clinical interviews (see exclusion criteria below). The participants were recruited with convenience sampling: they were reached through researchers’ and students’ contacts.

Exclusion criteria consisted of any known chromosomal or hormonal disorder; any current or past psychiatric or neurological disorder; known impaired vision; any medications with known psychotropic effects; luteal phase for women; a score of 2 or greater on the Kinsey scale [26].

### 2.2. MRI Acquisition

The fMRI paradigm consisted of 1 session of about 40 min. The participants underwent 3 fMRI runs with 3 different body and face appraisal tasks, described below; a high-resolution 3D-T1 weighted anatomical was acquired before the fMRI runs.

The MRI was acquired with 3 T scanner (Philips, Ingenia) equipped with a viewer and a joystick MRI compatible (VisuaStim Digital, Resonance Technology Inc., Northridge, CA, USA). The fMRI time series were acquired using a GE-EPI sequence with the following parameters: 34 slices, 4 mm of slice thickness, in-plane resolution of 2.15 mm × 2.15 mm, 3 initial dummy volumes discarded for signal steady-state, FA 90°, TR 3 s. We used 96, 144, and 168 volumes acquisitions. The anatomical scan was acquired with an MPRAGE equivalent sequence with the following parameters: 1 mm isotropic voxel, TE = 3.7 ms, TR = 8.09 ms.

### 2.3. fMRI Tasks and Stimuli

The tasks consisted of the visual observation of pictures and of, their judgment through button presses, choosing between two alternative evaluations: one positive and the other negative. All the stimuli were presented in a randomized order using dedicated software (PST, E-Prime).

**Figure 1:**
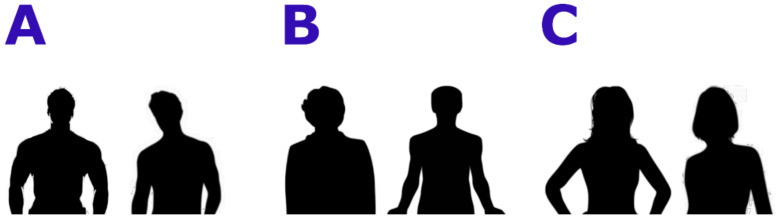
Examples of stimuli used during the Body Projection task. (**A**): male bodies; (**B**): neutral bodies, (**C**): female bodies.

In the first experiment, Body Projection, the stimuli consisted of black stylized bodies of full stand-up bodies with masculine or feminine or ephebic features; two examples for each category can be found in Figure 1 The bodies included heads and limbs, but no objects or clothes. Sexual secondary traits like breast, waist-to-hip, or waist-to-shoulder ratio were evident. Eight different bodies for every condition (male, female, androgynous (lacking clear sexual characterization)) were presented for a total number of 24 trials. Every trial consisted of the observation of a body (3 s), the presentation of the question “Hai una sensazione positiva o negativa su quanto hai appena visto?” (Translation: “Do you have a positive or negative feeling about what you have just seen?”) and the button press (choice yes/no represented by thumb up/down icons depicted over the position of the buttons on the joystick) for a maximum time of 9 s. A fixation cross (3 s) was presented before and after stimuli and questions, to help the correct orienting of the attention.

The instructions were: Imagine that the bodies that you will observe are the shadows that your body projects and, when asked, answer the questions as fast as you can.

In the second experiment, Static Morphing, the stimuli were images of the face of the subject, morphed with gender-specific face templates, using a morphing software (Psychomorph http://pics.psych.stir.ac.uk/ESRC/software.html, accessed on 1 September 2016 [27]), in order to obtain a feminization or a masculinization of the patient’s own face.

Participants’ faces were photographed with a fixed digital camera before the MRI session, with the same artificial light condition and exposure time in each photo session; they were identically positioned against a white wall. All the pictures were matched with the templates in terms of average luminance and RMS contrast (that is, standard deviation of luminance) using the SHINE toolbox for Matlab on the value (“V”) component of the HSV representation of the images.

**Figure 2:**
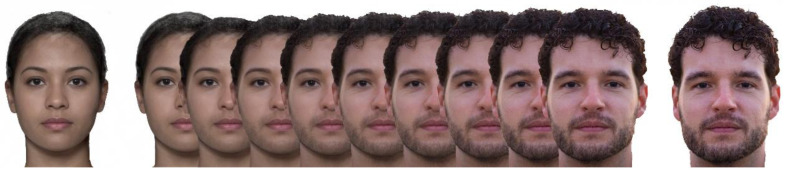
On the left, an average female template (DeBruine, Lisa & Jones, Benedict (2017), obtained using the app at http://faceresearch.org/demos/average (accessed on 1 January 2020), and shared with a CC-BY-4.0 license. On the right, a picture of a male model (not participant), sourced under a free license from unsplash.com. Between the faces, 9 equally spaced morphing steps, obtained using Webmorph (see article text). **The image is created to illustrate the experimental procedure and does not include stimuli used in this study, to respect participants’ privacy**.

The templates used to feminize or masculinize the subjects’ faces were obtained by averaging 32 men and women faces, downloaded from a shared database of faces photography (Chicago Face Database, CFD, http://chicagofaces.org, accessed on 1 September 2016). We chose images depicting Caucasian males and females in the same range of age as our subjects. Figure 2 shows an illustrative example realized using a female template and a free model male face obtained from a website (https://unsplash.com, accessed on 1 September 2016). Images used in the experiment are not shown in this paper, to comply with the regulations set by the Italian and European laws on privacy. For the experiment, we generated stimuli characterized by different percentages (0, 25, 50, 100%) of blending with the templates.

Eight different faces (masculinized or feminized 0, 25, 50, and 100%) were presented twice for a total number of 16 trials. Every trial consisted in the observation of a face (3 s), the presentation of two possible questions (random order) Hai una sensazione positiva o negativa su quanto hai appena visto? or Can you identify yourself in the image that you have just seen? followed by the button press (yes/no) for a maximum time of 9 s. A cross fixation (3 s) was presented before and after stimuli and questions.

The third experiment, Dynamic Morphing, was like experiment 2, with the difference that the stimuli consisted of short videos (6 s) showing gradually the morphing from the origin to the target picture. Twelve different videos (masculinized or feminized 25, 50, and 100%, origin from subject or template face) were presented for a total number of 24 trials. In this experiment’s instructions and questions, the word ‘face’ was changed to ‘video’.

### 2.4. Data Analysis

Data analysis was performed using FSL 6.0.3 (https://fsl.fmrib.ox.ac.uk, accessed on 1 December 2019) running on Centos 7 OS. The data were preprocessed as follows: motion correction (MCFLIRT), grand-mean intensity normalization of the entire 4D dataset by a single multiplicative factor, non-brain removal, 2-stage registration with MNI 152 template using T1-weighted images and functional data by combining two techniques: FLIRT (subject T1 into MNI 152 template) and BBR (subject functional data into subject T1 images). In addition, we applied a 5 mm spatial smoothing before the ICA-AROMA pipeline [28] which automatically identifies and removes motion-related components computed by the ICA approach. The data were modeled with FEAT (fMRI Expert Analysis Tool) using the general linear modeling (GLM, FILM pre-whitening, double gamma HRF convolution) for the first-level analysis. We were interested in the conditions of stimuli observation. In the first experiment we categorized the regressors as male, female or ephebic bodies (8 stimuli per condition); in the second experiment as own (0% morph), masculinized (25–50% male template), feminized (25–50% female template), other (100% morph) faces (4 stimuli per condition); in the third experiment as feminization (from own to female template), masculinization (from own to male template), defeminization (from female template to own face), demasculinization (from male template to own face) videos (6 stimuli per condition).

For second-level analysis (i.e., across subjects) we used FLAME (FMRIB’s Local Analysis of Mixed Effects) mixed effect to compare stimuli processing between gender groups (males vs. females). Z (Gaussianised T/F) statistic images were thresholded non-parametrically using clusters determined by Z > 2.3 and a (corrected) cluster significance threshold of *p* < 0.05 [29].

We compared the gender groups’ activations for the 3 experiments. To check if we can predict the gender from the individual brain activity we extracted GLM betas from 2 Region of Interest (ROI) in important areas of the ventral stream pathway for visual stimuli processing, in particular for faces and own face [30,31]: mainly the right primary and secondary visual cortices (such as V1, V2) and the right fusiform gyrus (FUS). The ROIs were delineated with the following method. First, we collected the betas for each experimental condition and merge them into a unique file. This file was organized in order to maintain the gender of the participants separated.

The betas were extracted for the following conditions: male body, female body, androgynous body (as control condition), own face, congruent face, incongruent face, congruent video morph (M to male template or F to female template), incongruent video morph, congruent inverse video morph (e.g.,: male template to M participant), incongruent inverse video morph (e.g.,: male template to F participant), (10 conditions per 2 ROIs = 20 betas). We computed univariate logistic regression using betas as independent variables and gender as dependent variable (20 different regressions), reporting for each the predictive power (computed as the Area Under the Receiver Operating Characteristic Curve—AUC), and p values associated with the univariate model. We also compared the AUC of models obtained using V1 betas with models computed using FUS betas by means of t-tests. The same approach was used to compare models built on the betas of static vs. dynamic stimuli within each ROI.

Behavioral responses were analyzed by computing the frequencies of the positive answers (thumb-up button press). The average frequency was computed for each experiment and subject in two conditions: congruent (the sex of the stimulus equal to the sex of the subject) or incongruent (sex of the stimulus different from the sex of the subject). For experiment 1 the sex of the stimulus was inferred from the body (we discarded the trials with androgynous silhouettes from the analysis), for experiments 2 and 3 the sex of the stimulus was taken to be equivalent to the template adopted (so, a female face feminized, and a male face feminized would both considered as a ‘female’ stimulus). To assess the difference between the two conditions, we conducted non-parametric tests (Wilcoxon rank-sign tests) for the three experiments separately. All the statistical analyses, excluding fMRI data, were conducted using SAS Studio OnDemand (version 3.8, SAS Institute Inc., Cary, NC, USA).

## 3. Results

After quality inspection (motion and MR-related artifacts) we removed 2 females and 1 male from the video experiment and no subjects from experiments 1 and 2.

The activations for the male and female groups and different experiments and conditions are shown in Supplementary Figures S1–S11. In general, a widespread activation can be observed, generally bilateral, with some asymmetry and more areas active in the right hemisphere (experiments 2 and 3). The network of activations involved areas of the ventral visual pathway (occipital and temporal cortices, fusiform gyrus) and the dorsal attention areas (frontal eye fields, parietal lobes). Notably, the activation networks include areas that have been linked to the processing of faces and bodies (EBA and FBA). Nevertheless, specific differences can be observed in different experiments across groups.

**Figure 3:**
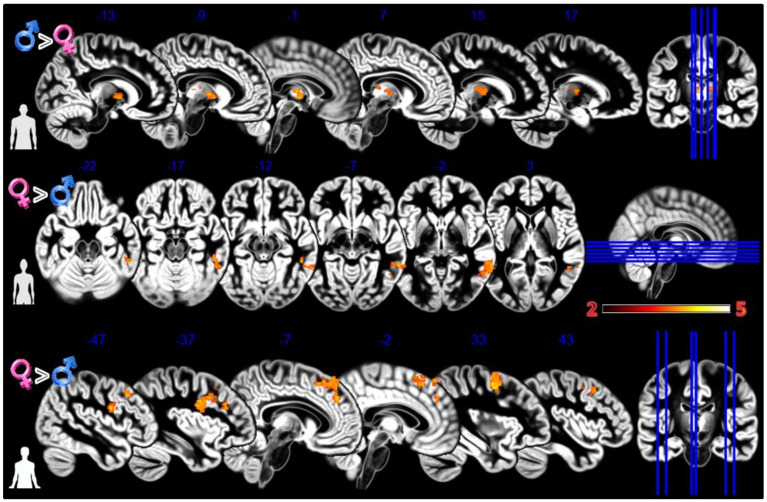
Group differences for the Body Projection experiment. First row Males greater than Females for male bodies, second row Females greater than Males for female bodies, and third row Females greater than Males for asexual bodies. In red significant clusters (*p* corr < 0.05) on a gray matter ICBM brain template, neurological convention, color scale in the bar maps for Z scores. In blue MNI coordinates in mm, and lines to show the slice positions on the orthogonal projections (right side of the figure).

In the first experiment, Body Projection (Figure 3, Table 1), the male group activated more the bilateral thalamus when looking at sex congruent body images; the female group activated more the middle and inferior temporal gyrus when looking at sex congruent stimuli and the frontal superior medial cortex and bilaterally the more anterior and lateral portions of the precentral cortex when looking at androgynous body images.

**Figure 4:**
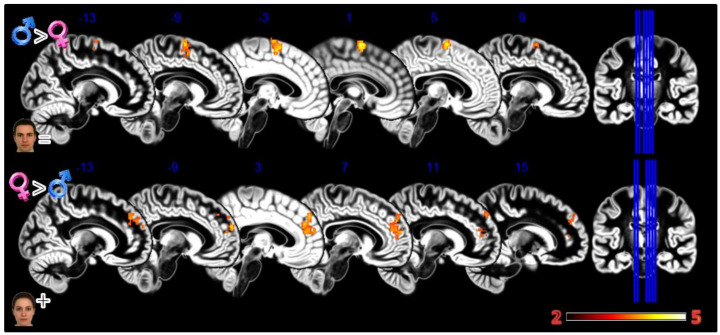
Group differences for the Static Morphing experiment. First row Males greater than Females for looking at own face, second row Females greater than Males for looking at feminized faces. In red significant clusters (*p* corr < 0.05) on a gray matter ICBM brain template, neurological convention, color scale in the bar maps for Z scores. In blue MNI coordinates in mm, and lines to show the slice positions on the orthogonal projections (right side of the figure).

In the second experiment, Static Morphing (Figure 4, Table 1), the male group activated more the supplementary motor area (SMA) when looking at their own face; the female group activated more the dorsomedial prefrontal cortex (dmPFC).

**Figure 5:**
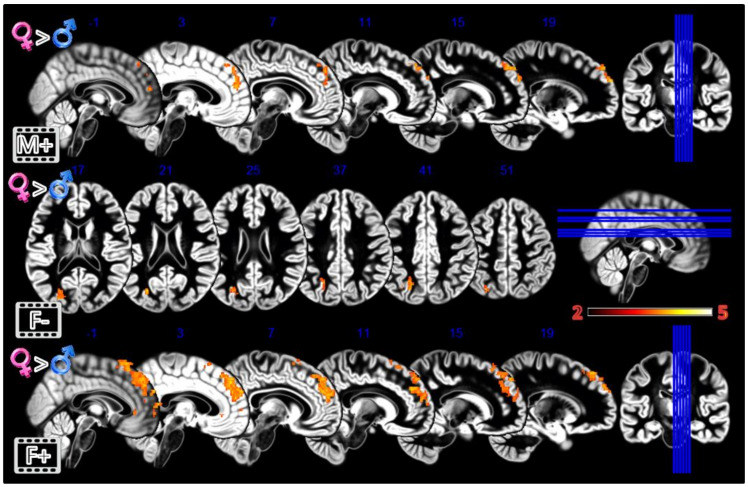
Group differences for the Dynamic Morphing experiment. All the rows Females greater than Males for masculinization (**first row**), defeminization (**second row**) and feminization (**third row**) morphings. In red significant clusters (*p* corr < 0.05) on a gray matter ICBM brain template, neurological convention, color scale in the bar maps for Z scores. In blue MNI coordinates in mm, and lines to show the slice positions on the orthogonal projections (right side of the figure).

In the third experiment, Dynamic Morphing (Figure 5, Table 1), the female group activated more the dmPFC when observing both observing the feminization or the masculinization of their face, but with a more widespread activation (2804 vs. 448 voxels) for congruent sex condition. Also, the defeminization produced more activations in females compared to males, but in the left superior parietal lobule and middle occipital gyrus.

The analysis carried out on the responses given to the trials showed a significant effect of the congruency factor for all three experimental tasks: bodies (Z = 3.218; *p* < 0.001; r = 0.75), faces (Z = 2.191; *p* < 0.001; r = 0.51), videos (Z = 2.943; *p* < 0.001; r = 0.69). In all cases, congruent stimuli received positive feedback more often than incongruent stimuli: body (congruent = 67%, incongruent = 60%), faces (congruent = 72%, incongruent = 54%), videos (congruent = 37%, incongruent = 14%).

The univariate logistic regressions (see Table 2) demonstrated that 12 betas were significant in models for predicting the sex of an individual from brain activations.

The regression that was more accurately discriminated between sexes were derived from betas extracted from V1 activations in the Static Morphing experiment, even if only 4 out of 10 V1 regressions were significant, while 8 out of 10 FUS regressions reached the statistical threshold for significance. There’s no overall difference between the mean AUC of V1 and FUS models (t(9) = 0.43, *p* = ns), but the AUC of V1 models when observing the static morphed stimuli is higher than the one of FUS models (t(2) = 6.9, *p* = 0.02), which is particularly noteworthy as we are comparing two groups of three observations each. This is confirmed by noting that, using only the V1 ROI betas, models using static stimuli performed better than models that used dynamic stimuli (t(8) = 3.04, *p* = 0.015), while no such difference was found using the FUS ROI betas

## 4. Discussion

Alongside other neuroanatomical techniques, fMRI has been used in previous studies to investigate dimorphism and differential patterns of activation between males and females. These studies used standard stimuli such as verbal fluency, mental rotation and motivational tasks [16,17,18], but no specific tasks have been at the moment developed to be used as stimuli to investigate processing streams that link self-perception and self-awareness with gender identity.

Expanding over previous studies, this paper explicitly seeks to fill this gap and aims both at verifying if gender-linked differences in brain activity can be studied by a visual stimulation involving the gender features of the body image of the subject. We also aimed at validating an fMRI protocol that could form the basis for further studies investigating sexual dimorphism, and to expand and validate previous results obtained by investigating self-directed gender attribution, as previously done by [15].

This exploratory study was limited to participants whose biological sex and gender were congruent. Likewise, to avoid introducing confounding variables, we enrolled only heterosexual participants (Kinsey scale score < 2).

We observed in some conditions of all the experiments a greater activity in the dmPFC for the female group (observation of androgynous bodies, static feminization, dynamic masculinization, and feminization). Given the nature of the task, and the explicit presence of questions related to self-identification with the stimuli, it is likely that self-processing mechanisms are involved in the activations found by this study. In fact, dmPFC activity has been linked to self-identity [32,33] and self-identity suppression during acting performances or in psychological dissociation [33,34]. More simply, dmPFC activations can be linked to the regulation of the emotional reaction to the stimuli [35]. We also observed an anterior/posterior difference in the activations generated by different stimuli: dynamic and feminized stimuli activated more voxels situated more anteriorly/ventrally (Table 1, Figure 2, Figure 3 and Figure 4) compared to static and asexual stimuli. This can be linked to the functional topography of dmPFC where the posterior-anterior direction corresponds to an increment in abstraction/complexity [36] and dorsal-ventral areas dichotomy between appraisal and regulation functions [37]. Furthermore, it has been proposed [8,38] that ventral areas of the PFC might be involved in the attribution of significance to self-related stimuli, showing activations that scale with personal importance attributed to self-representations. Such a theory might explain why females activated more ventral voxels when observing feminized stimuli rather than androgynous bodies. It is also worth noting that the PFC, and especially the dmPFC, is an important player in the ability to attribute mental states to oneself and others, the Theory of Mind (ToM). This ability can be elicited by the appraisal tasks, especially because of the need to access the present-self representation [39]. Females have often been described as more social, empathic, and skilled in ToM [40,41,42], suggesting another explanation for the inter-gender differences in activations observed in the present study.

Females also activated more the middle and inferior temporal gyrus during the observation of female bodies, areas associated with visual stimuli processing, recognition and analysis [43]. However, we were not able to identify differential activation between the genders when considering either EBA or FBA. As these two areas, too, have been linked to the self-other differentiation [6], it is likely that the different brain activations are not linked to gender-specific differences in identification with the stimuli, at least when using silhouettes. It is however worth remembering that, given our small sample size, the absence of evidence cannot be construed as proof of the absence of a difference between the two groups. Finally, in the female group, the superior parietal lobule was more active when looking at the dynamic de-feminization an area observed in different studies that used complex visual face stimuli of self and others [44,45].

The male group demonstrated more activation in the thalamus [46] during the observation of male bodies. The thalamus is a subcortical hub involved in many different processes, from sensory to motor; it also contributes to regulating sleep, alertness and consciousness. It is difficult to speculate what specific subnuclei were included, but, broadly speaking, it seems that the activation was more anterior-medial, a portion usually considered to be involved in attention and memory and connected to the limbic system [46]. The male group also activated more the supplementary eye field, a portion of the SMA that contributes to visual search and is important for visual salience [47].

These results are partially at odds with the ones reported by Majid and collaborators [15] who used a sample of 15 male and 15 female participants and investigated the neural substrates of own and other’s body perception, and only reported areas in which male participants’ activations are larger than females. In contrast, despite our smaller sample size, we observe areas of greater activation in the female group, when they observed androgynous bodies. The areas include the precentral gyri, whose role in body ownership tasks has been confirmed by a meta-analysis by Salvato and collaborators [43], and the superior frontal gyrus, which has been indicated as involved in the distinction between self, familiar and unfamiliar bodies [34].

We also note that, in this study, differences in activations were more likely to be found in areas on the left hemisphere than on the right, as expected given the existing literature [25]. In particular, the left dmPFC was shown to be differentially activated (more in women than in men) in all three tasks: body projection, static morphed faces and dynamic morphed faces. As small sample sizes tend to highlight large differences, we suggest that this area of the brain might be of particular interest for future studies that want to investigate the difference between the two genders in self-identification mechanisms.

Finally, the behavioral analysis showed that the congruent condition between participants’ biological sex and gender was a significant factor, as participants on average disliked the incongruence between their identity and stimuli manipulated towards the opposite sex.

As an additional step, we used logistic regressions to determine whether the brain activation in areas involved with different aspects of visual processing could be used to reliably distinguish male from female participants in this task. This was principally done as a preliminary step before expanding the paradigm used in this paper to other groups of participants, for instance to participants’ gender dysphoria (specifically, Male-to-female, following, for instance, the study by [48]). Interestingly, all classifiers based on single ROIs performed better than random classifiers. Overall, no predictive difference could be found between FUS and V1 models, but no models computed from data extracted from the V1 ROI were significant when the stimuli were dynamic (morphing animations), while this was not true for the FUS-based models. This might reflect the different roles in the visual processing of the two areas, as the fusiform gyrus lies at a later processing stage with respect to V1, and has sub-areas specific for both bodies (Ref. [49] and faces [10]). Furthermore, it has been shown that this area could have a different neural response to the self and others, at least when participants are discriminating between their own and others’ bodies [6,50]. While this study supports the findings of the literature, we extend them, as the models based on the fusiform gyrus were able to predict the gender of the subjects even when considering face-based tasks.

### Strengths and Limitations

This is the first study focused on exploring the differential brain activation patterns elicited in males and females by manipulating the sexual characteristics of the stimuli (faces and bodies). While we provided evidence that the protocol we proposed can highlight gender-specific differences, including converging evidence of the role played by the dmPFC, some limitations should be acknowledged. First, in this study we enrolled a small group. This was justified by the scope of this work—that is, the first step of validating a novel paradigm idea and understanding what factors could contribute more to the differentiation between sexes in self-other discrimination and rating tasks. Furthermore, in this experiment it is difficult to assess the cognitive effects of sex hormones, even if we tried to limit the state effects and focus on trait effects of hormones by excluding the females in the luteal phase (we controlled this factor by only enrolling females within two weeks from last menses). Finally, further directions of the current research could investigate the responses to the paradigm in Male to Female and Female to Male DG subjects, focusing on the differential role of the dmPFC and of the activations shown to be able to predict the gender of the respondent with greater accuracy.

## Figures and Tables

**Table 1:** Group differences for different experiments and conditions.

Cluster	Voxels	*p*	Z_max_	X Y Z [mm]	Area	BA
Experiment 1, M > F (Male Bodies)						
1	474	0.005	3.55	−8 −8 4	L Thalamus	-
Experiment 1, F > M (Female Bodies)						
1	439	0.012	3.94	62 −44 −6	R Middle Temporal	22
Experiment 1, F > M (Asexual Bodies)						
1	630	0.00067	3.67	−38 −4 26	L Precental	6
2	490	0.00434	3.55	−10 42 54	L Frontal Medial Sup	9
3	433	0.00975	3.7	34 −2 52	R Precentral	6
Experiment 2, M > F (Own Face)						
1	474	0.00704	4.91	2 −2 −68	R SMA	6
Experiment 2, F > M (Feminized Face)						
1	403	0.0161	4.26	−10 62 22	L Frontal Medial Sup	10
Experiment 3, F > M (Feminized Video)						
1	2804	<0.00001	4.17	−6 38 52	L Frontal Medial Sup	8
Experiment 3, F > M (Masculinized Video)						
1	448	0.0105	3.46	18 62 28	R Frontal Sup	10
Experiment 3, F > M (Demasculinized Video)						
1	395	0.0228	3.68	−22 −62 38	L Parietal Sup	7

F = female, M = male, Sup = superior, Inf = inferior, SMA = Supplementary Motor Area, L = left, R = right, BA = Brodmann Area, Area of the peak activations classified with automated anatomical labeling atlas.

**Table 2:** Logistic regression results using beta regressors.

ROIs	Experiment	Stimuli	AUC ROC	*p*
FUS	Body	Congruent	0.812	**0.028**
FUS	Body	Incongruent	0.756	**0.047**
FUS	Body	Neutral	0.757	**0.038**
FUS	Video morphing	Self to congruent template	0.778	**0.043**
FUS	Video morphing	Self to incongruent	0.778	**0.025**
FUS	Video morphing	Congruent template to self	0.854	**0.02**
FUS	Video morphing	Incongruent to self	0.765	**0.041**
FUS	Static morphing	Congruent	0.736	**0.035**
FUS	Static morphing	Incongruent	0.694	0.065
FUS	Static morphing	Neutral (self)	0.618	0.396
V1	Body	Congruent	0.84	**0.024**
V1	Body	Incongruent	0.722	0.11
V1	Body	Neutral	0.763	**0.052**
V1	Video morphing	Self to congruent template	0.701	0.102
V1	Video morphing	Self to incongruent	0.769	0.055
V1	Video morphing	Congruent template to self	0.645	0.187
V1	Video morphing	Incongruent to self	0.701	0.102
V1	Static morphing	Congruent	0.861	**0.014**
V1	Static morphing	Incongruent	0.84	**0.014**
V1	Static morphing	Neutral (self)	0.819	**0.012**

V1 = primary visual cortex, FUS = fusiform gyrus. Bold marks significant models (*p* < 0.05)

## Data Availability

Data is available to all scientists upon request sent to Federico D’Agata (federico.dagata@unito.it).

## Supplementary Materials

The following are available online at https://www.mdpi.com/article/10.3390/tomography8040176/s1, Figures S1–S11: Male and Female brain activations (Experiments 1, 2 and 3)
