# Supplementary material for: Investigation of Brain Activation Patterns Related to the Feminization or Masculinization of Body and Face Images across Genders
Source: Tomography. 2022 Aug 22;8(4):2093–106. doi: 10.3390/tomography8040176 (PMC9416062; doi:10.3390/tomography8040176)
Supplement: Supplementary file 1 [file tomography-08-00176-s001.zip › tomography-1799609-supplementary.pdf]

Supplementary Materials

fMRI correlates of the perception of feminized and masculinized body and face images across genders

Ceruti C*[1,], Cicerale A*^[2,3], Diano M[3], Sibona M[1], Motta G[,5], Crespi CM[,6], Gualerzi A[,7], Lanfranco F[,5], Bergui M[2], D’Agata F[2]

On behalf of the CIDIGeM Study Group - Center for Gender Dysphoria of Città della Salute e della Scienza Hospital, Turin, Italy.

Supplementary Figures

Supplementary Figure S1. Experiment 1, Body Projection, Male Bodies

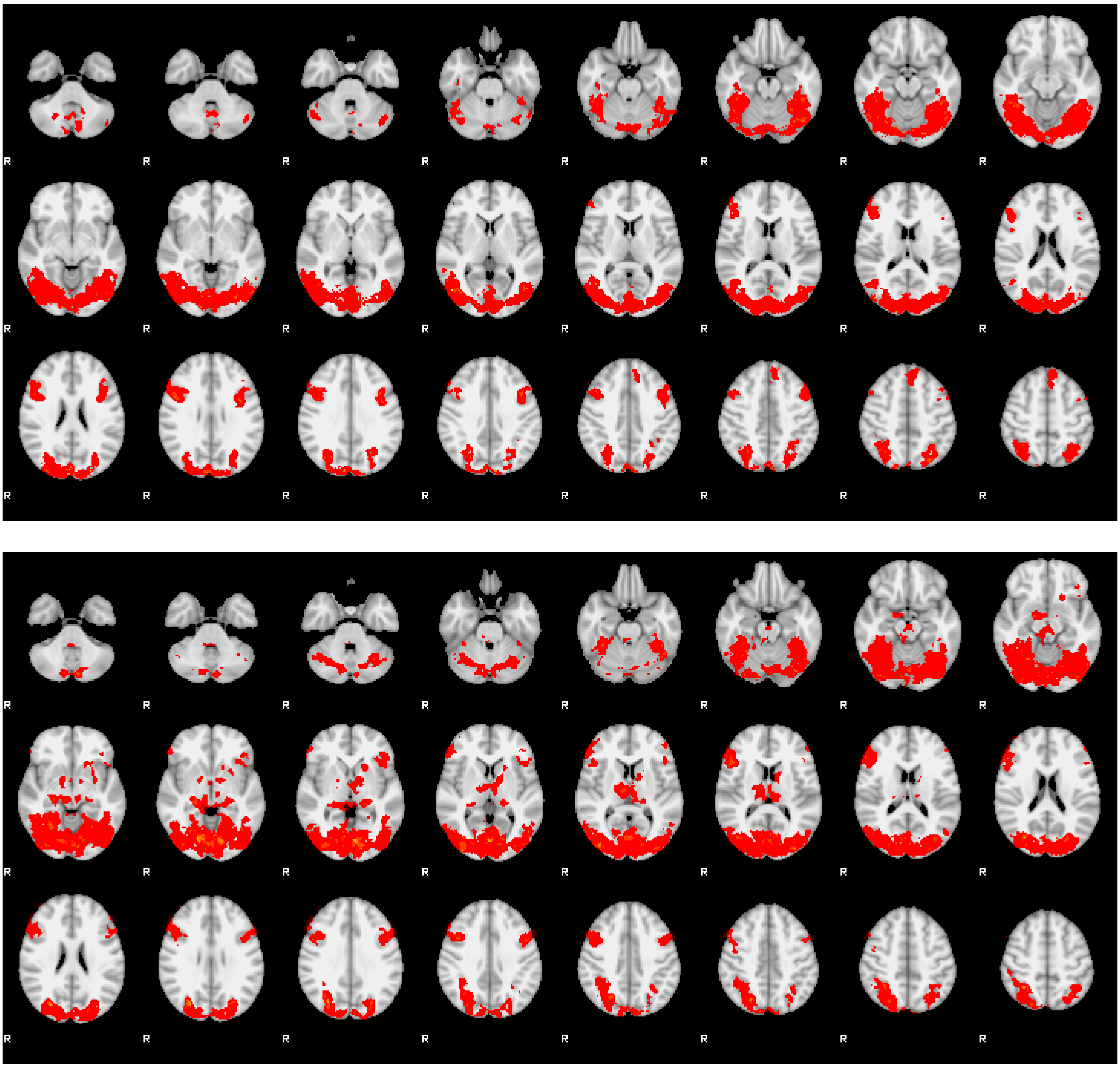

Top = Female activations, Bottom = Male activations, p<.05 cluster corrected, R = right

Supplementary Figure S2. Experiment 1, Body Projection, Female Bodies

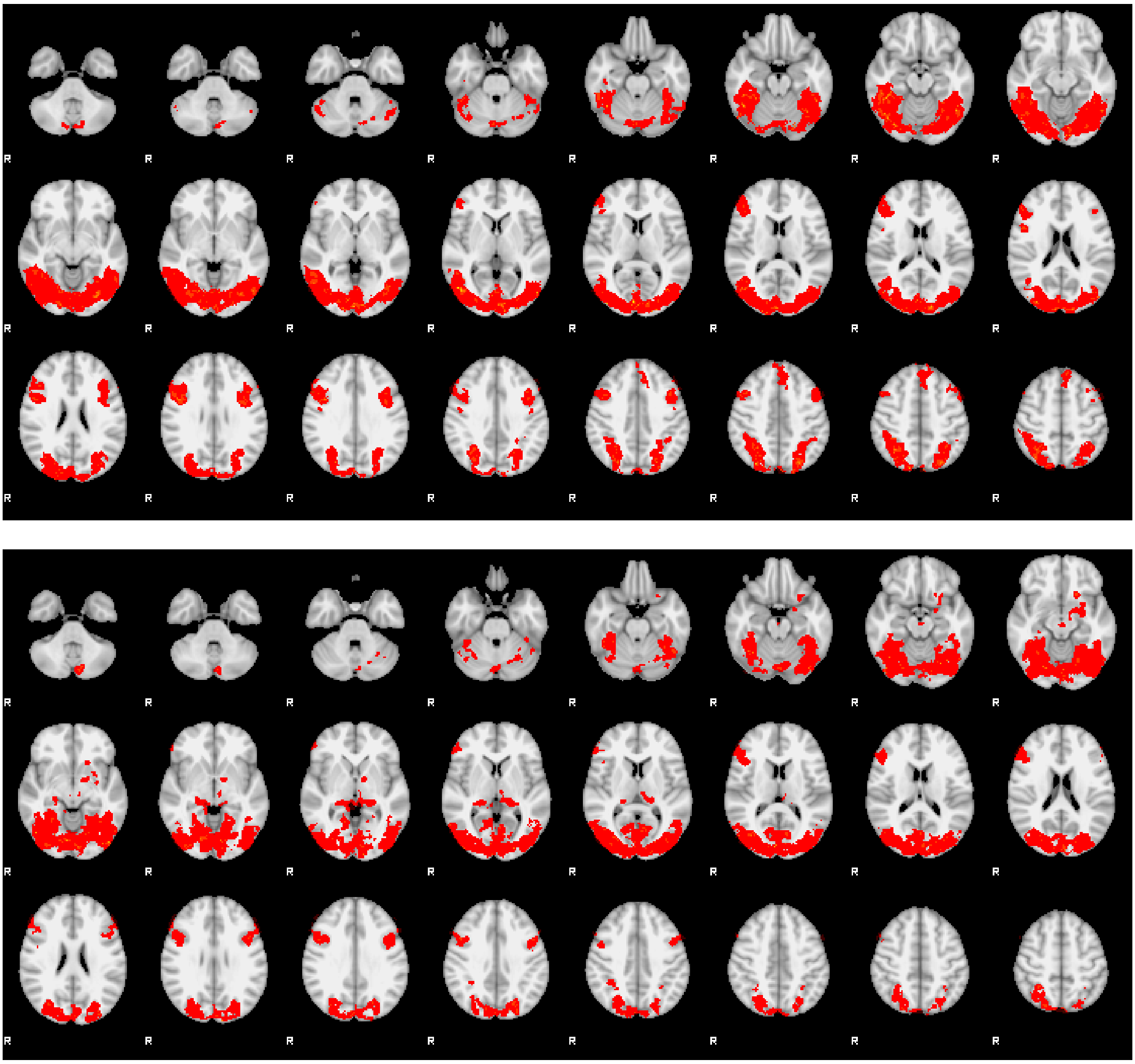

Top = Female activations, Bottom = Male activations, p<.05 cluster corrected, R = right

Supplementary Figure S3. Experiment 1, Body Projection, Asexual Bodies

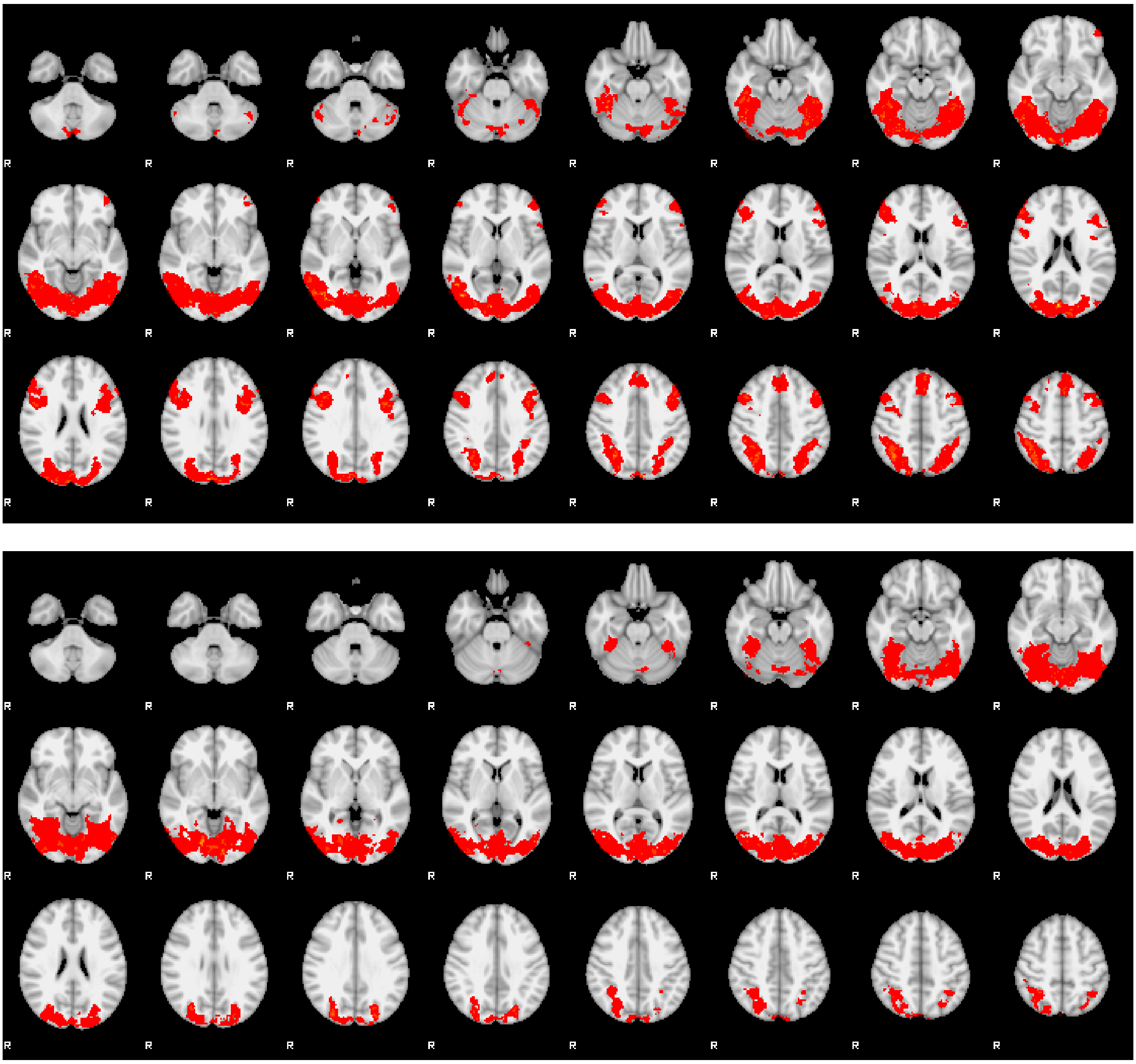

Top = Female activations, Bottom = Male activations, p<.05 cluster corrected, R = right

Supplementary Figure S4. Experiment 2, Static Morphing, Own Face

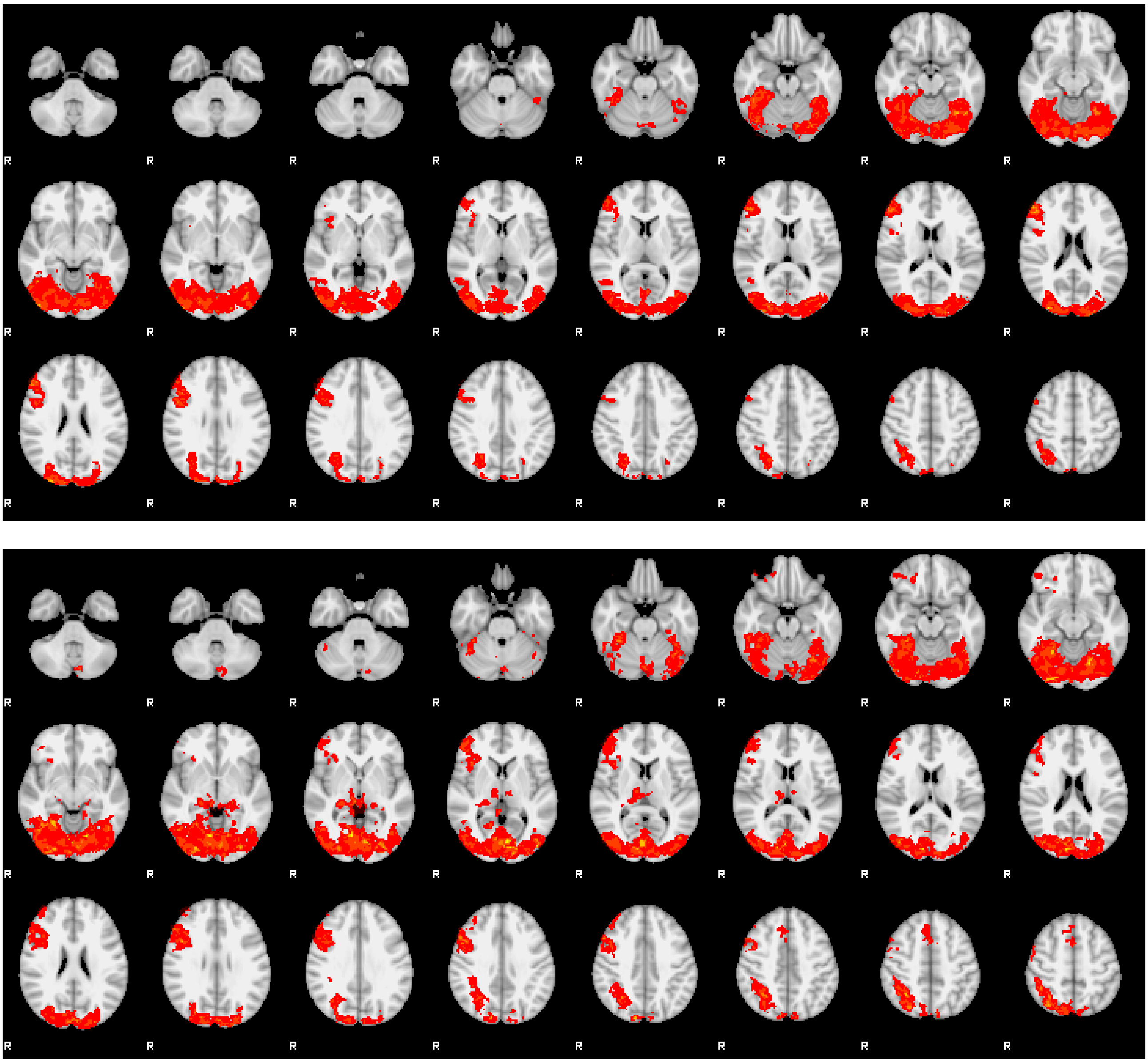

Top = Female activations, Bottom = Male activations, p<.05 cluster corrected, R = right

Supplementary Figure S5. Experiment 2, Static Morphing, Feminized Face

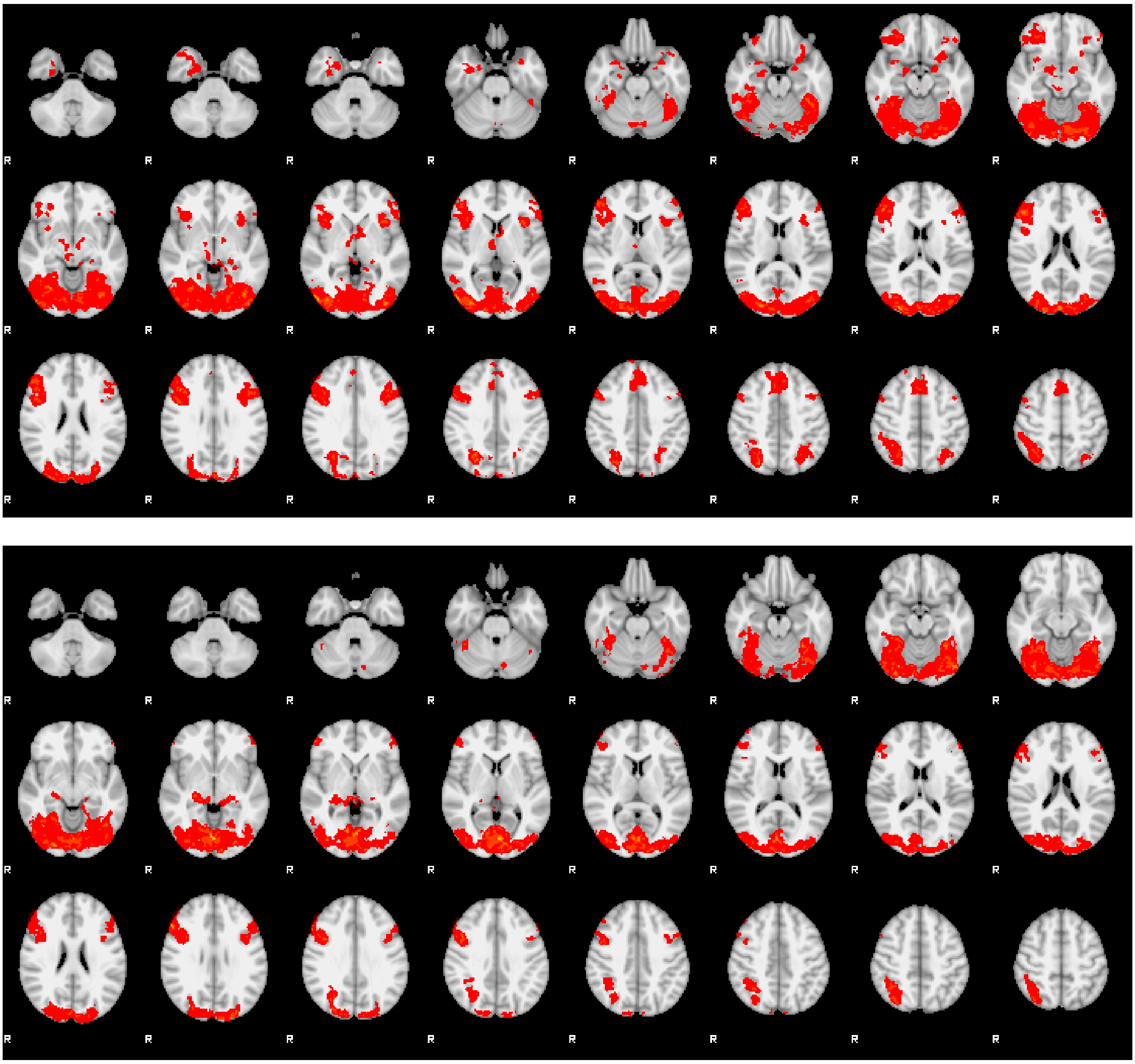

Top = Female activations, Bottom = Male activations, p<.05 cluster corrected, R = right

Supplementary Figure S6. Experiment 2, Static Morphing, Masculinized Face

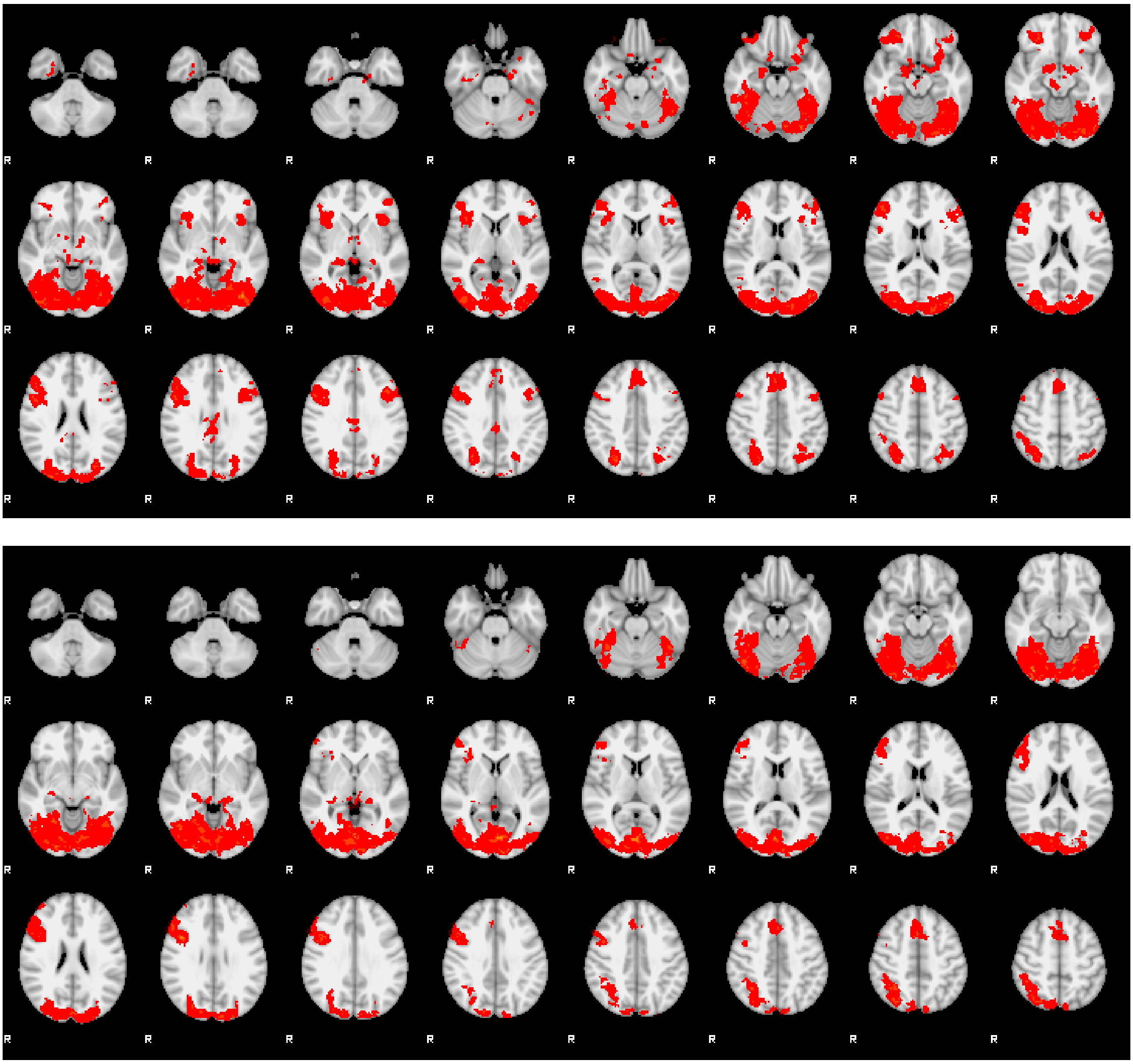

Top = Female activations, Bottom = Male activations, p<.05 cluster corrected, R = right

Supplementary Figure S7. Experiment 2, Static Morphing, Other Face

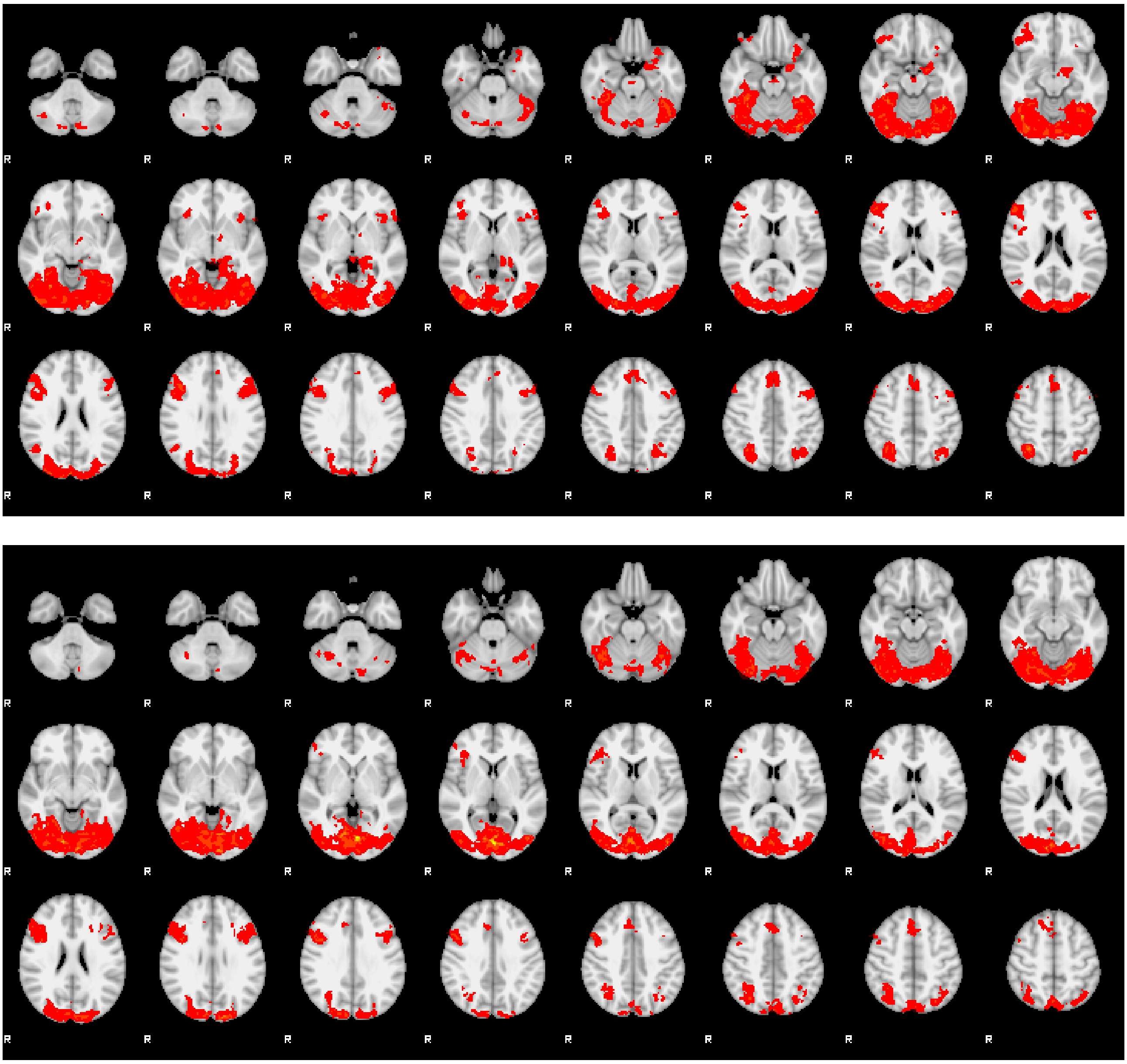

Top = Female activations, Bottom = Male activations, p<.05 cluster corrected, R = right

Supplementary Figure S8. Experiment 3, Dynamic Morphing, Masculinization Video

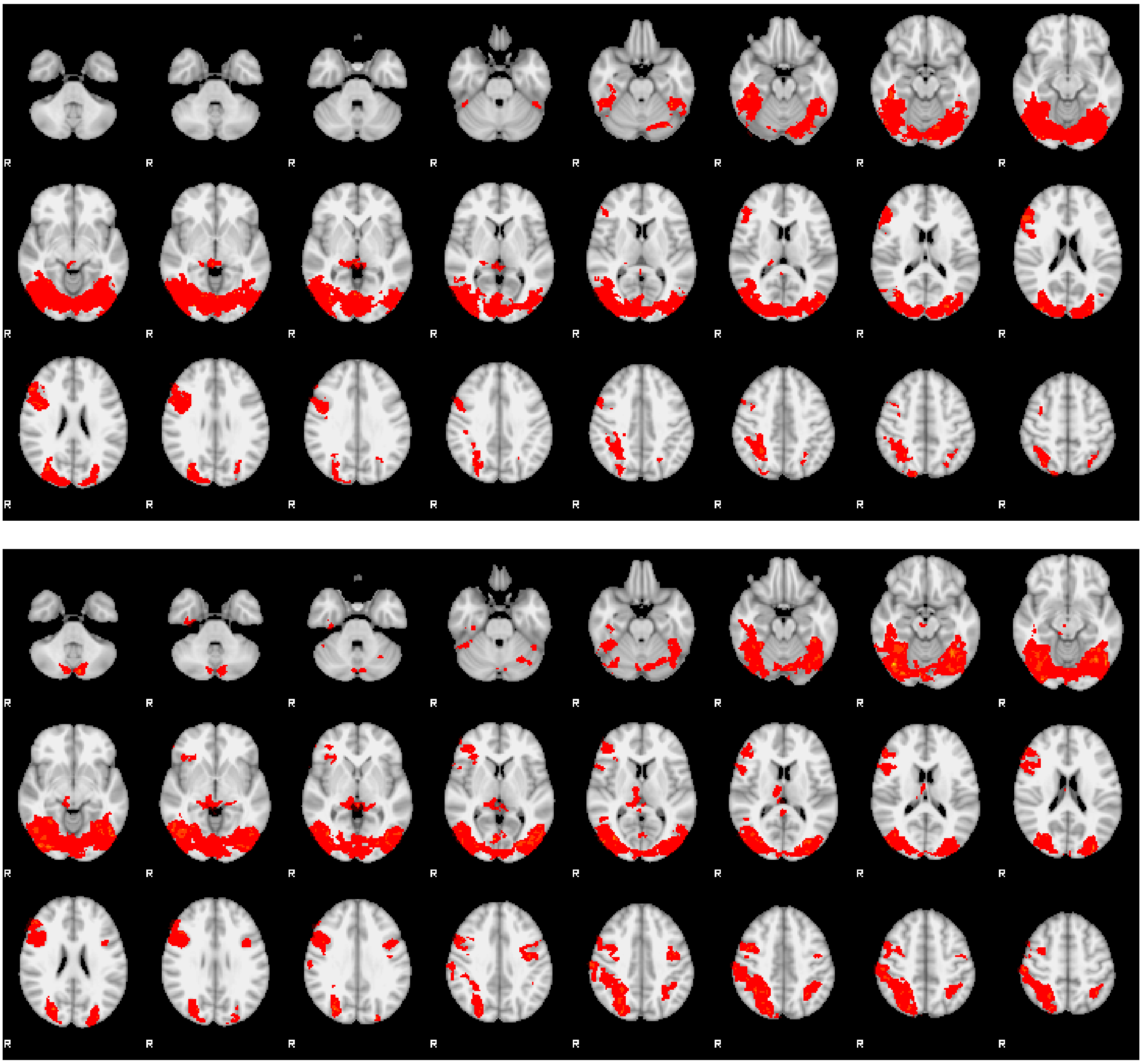

Top = Female activations, Bottom = Male activations, p<.05 cluster corrected, R = right

Supplementary Figure S9. Experiment 3, Dynamic Morphing, Feminization Video

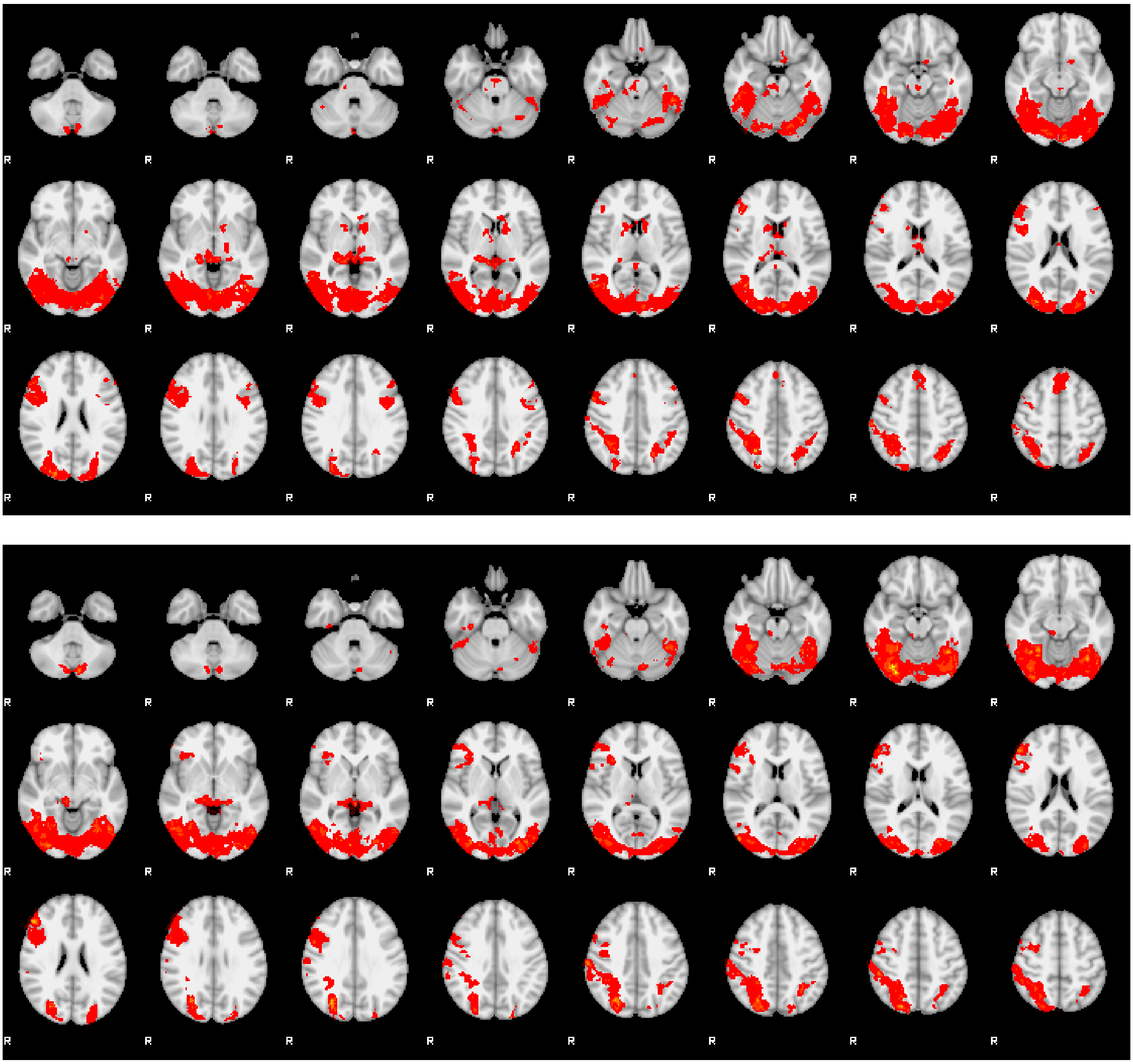

Top = Female activations, Bottom = Male activations, p<.05 cluster corrected, R = right

Supplementary Figure S10. Experiment 3, Dynamic Morphing, Demasculinization Video

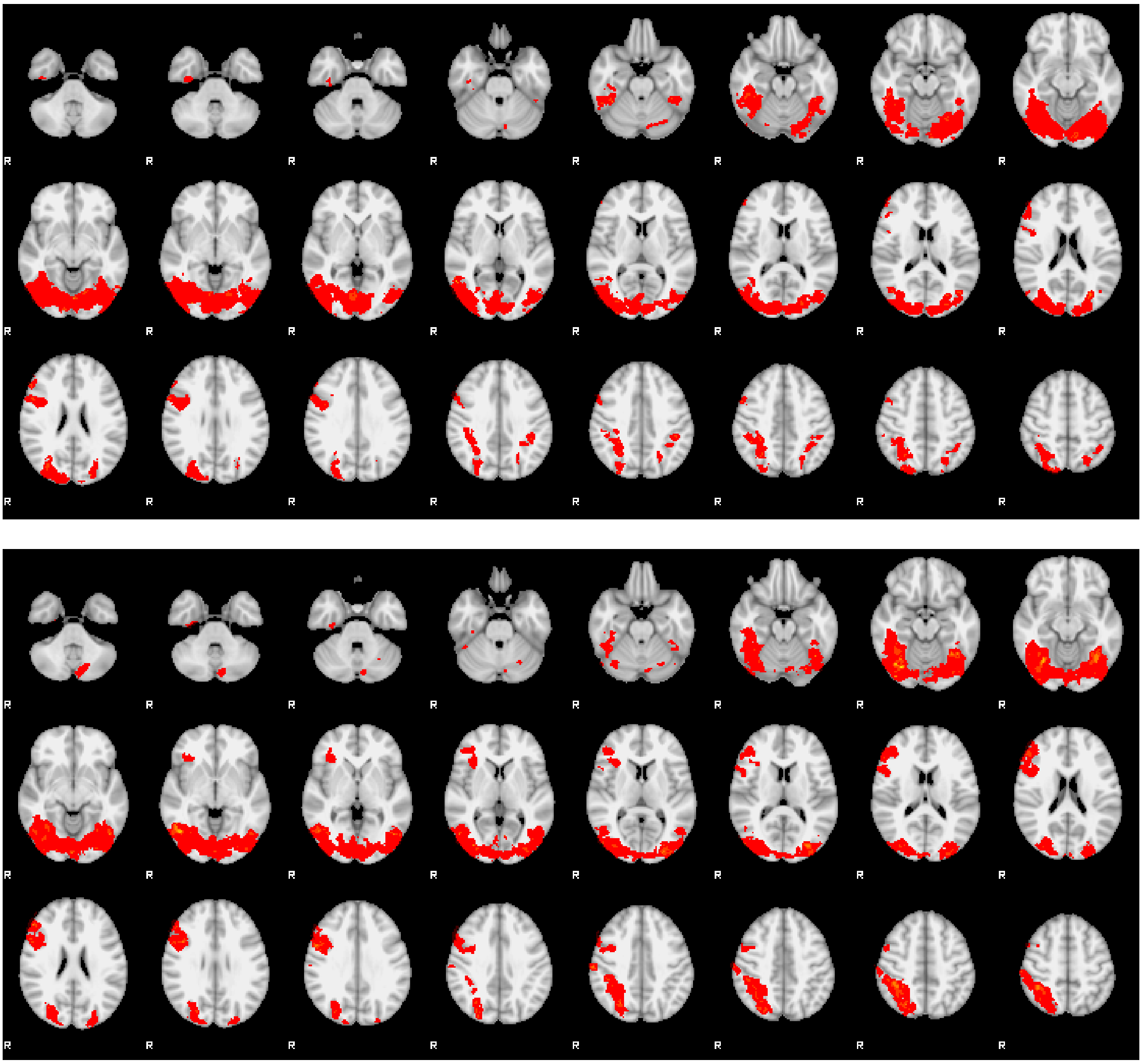

Top = Female activations, Bottom = Male activations, p<.05 cluster corrected, R = right

Supplementary Figure S11. Experiment 3, Dynamic Morphing, Defeminization Video

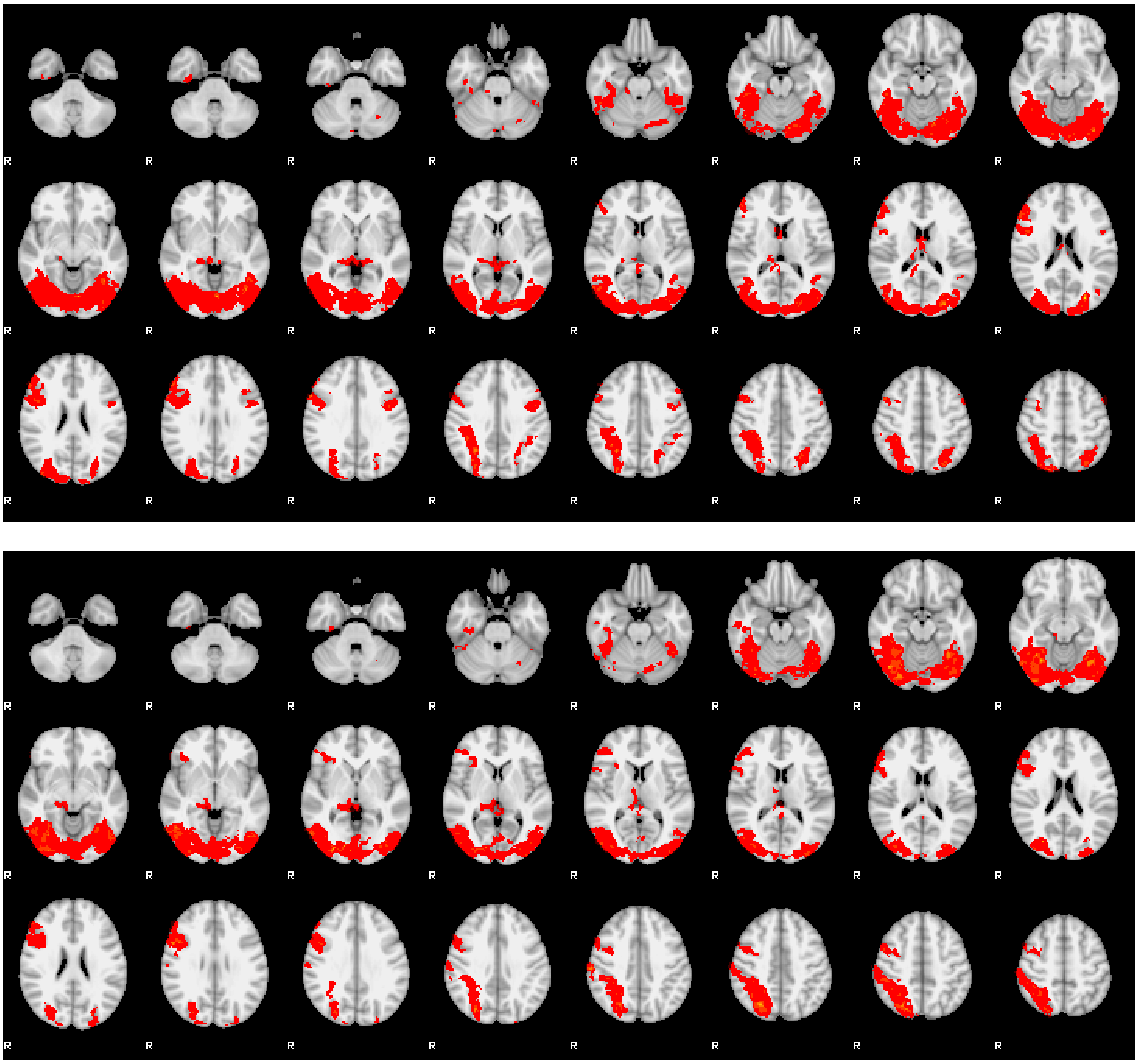

Top = Female activations, Bottom = Male activations, p<.05 cluster corrected, R = right
